# Contrasting clinical outcomes and socio‐economic impact of young versus elderly‐onset oral squamous cell carcinoma, a novel health economic analysis

**DOI:** 10.1002/cam4.6747

**Published:** 2024-01-15

**Authors:** Manraj Singh, Krishnakumar Thankappan, Deepak Balasubramanian, Vijay Pillai, Vivek Shetty, Vidyabhushan Rangappa, Naveen Hedne Chandrasekhar, Vikram Kekatpure, Moni Abraham Kuriakose, Arvind Krishnamurthy, Arun Mitra, Arun Pattatheyil, Prateek Jain, Subramania Iyer, N. Gopalakrishna Iyer, Narayana Subramaniam, the Head and Neck Cooperative Oncology Group

**Affiliations:** ^1^ Department of Head and Neck Surgery Singapore General Hospital and National Cancer Centre Singapore Singapore; ^2^ Department of Head and Neck Surgical Oncology Amrita Institute of Medical Sciences Kochi India; ^3^ Department of Head and Neck Surgical Oncology Mazumdar Shaw Medical Centre, Narayana Health Bangalore India; ^4^ Department of Surgical Oncology Cancer Institute (WIA) Chennai India; ^5^ Department of Head and Neck Surgical Oncology Tata Medical Centre Kolkata India; ^6^ Present address: Department of Head and Neck Surgical Oncology Apollo Proton Cancer Centre Chennai India; ^7^ Present address: Department of Head and Neck Surgical Oncology Cytecare Hospital Bangalore India; ^8^ Present address: Department of Head and Neck Surgical Oncology Sri Shankara Cancer Hospital and Research Centre Bangalore India

**Keywords:** head and neck cancer, health economics, oral cancer

## Abstract

**Objectives:**

The incidence of young‐onset oral squamous cell carcinoma (OSCC) is growing, even among non‐smokers/drinkers. The effects of adverse histopathological features on long‐term oncologic outcomes between the young and old are controversial and confounded by significant heterogeneity. Few studies have evaluated the socio‐economic impact of premature mortality from OSCC. Our study seeks to quantify these differences and their economic impact on society.

**Materials and Methods:**

Four hundred and seventy‐eight young (<45 years) and 1660 old patients (≥45 years) with OSCC were studied. Logistic regression determined predictors of recurrence and death. Survival analysis was calculated via the Kaplan–Meier method. A separate health economic analysis was conducted for India and Singapore. Years of Potential Productive Life Lost (YPPLL) were estimated with the Human Capital Approach, and premature mortality cost was derived using population‐level data.

**Results:**

Adverse histopathological features were seen more frequently in young OSCC: PNI (42.9% vs. 35%, *p* = 0.002), LVI (22.4% vs. 17.3%, *p* = 0.013) and ENE (36% vs. 24.5%, *p* < 0.001). Although 5‐year OS/DSS were similar, the young cohort had received more intensive adjuvant therapy (CCRT 26.9% vs. 16.6%, *p* < 0.001). Among Singaporean males, the premature mortality cost per death was US $396,528, and per YPPLL was US $45,486. This was US $397,402 and US $38,458 for females. Among Indian males, the premature mortality cost per death was US $30,641, and per YPPLL was US $595. This was US $ 21,038 and US $305 for females.

**Conclusion:**

Young‐onset OSCC is an aggressive disease, mitigated by the ability to receive intensive adjuvant treatment. From our loss of productivity analysis, the socio‐economic costs from premature mortality are substantial. Early cancer screening and educational outreach campaigns should be tailored to this cohort. Alongside, more funding should be diverted to genetic research, developing novel biomarkers and improving the efficacy of adjuvant treatment in OSCC.

## 1 INTRODUCTION

Squamous cell carcinoma is the most common malignancy of the head and neck region, accounting for 90% of all cancers.^1^ Chronic exposure to oral mucosal carcinogens such as alcohol, tobacco and betel nut have been classically described as causative factors.^2^ Despite being a disease affecting mostly males in the fifth to eighth decades of life, there has been a notable epidemiological shift towards early‐onset cancer, particularly among non‐smokers.^3^, ^4^ The etiology behind this shift remains multifaceted, with hypotheses ranging from an altered immune microenvironment, differences in cellular processing of carcinogens, poor dentition and Human Papillomavirus (HPV) as possible causes.^5^, ^6^, ^7^, ^8^ The growing incidence of oral squamous cell carcinoma (OSCC) in the young devoid of traditional risk factors poses a significant public health concern, especially in countries such as India where prevalence is high. Several studies argue that younger patients with OSCC have an aggressive clinical course with poorer prognosis; although heterogeneity among subsites, age cut‐offs and datasets have hindered broad generalisations.^9^ In response, we endeavoured to study a large multicentric cohort of patients from Singapore and India. The aim was to scrutinise survival trends among both young and older patients (those over 45 years), identifying disparities in clinicodemographics, adverse prognostic features and the intensity of adjuvant treatment received. Moreover, we studied the interplay of these factors and their impact on survival and recurrence. Recognising that disease morbidity and reduced life expectancy inevitably impact a patient's economic productivity, particularly among younger individuals within the workforce, our study extends to incorporate a health economic analysis. This innovative approach, never before undertaken in these regions, aims to objectively quantify the cost of premature mortality from early‐onset disease. We anticipate that this study will shed light on the burgeoning prevalence of OSCC among the young, thereby influencing early national screening efforts and campaigns aimed at discouraging betel nut chewing, tobacco and alcohol consumption.

## 2 MATERIALS AND METHODS

### 2.1 Study population

Two thousand one hundred and thirty‐eight patients from a prospectively maintained database were studied. These comprised patients with biopsy‐proven OSCC treated with curative intent at five tertiary care institutions—National Cancer Centre (Singapore), Amrita Institute of Medical Sciences, Mazumdar Shaw Medical Centre, Tata Medical Centre and Cancer Institute Adyar (India) between 2006 and 2013. All patients included were treatment‐naïve. The primary tumour was completely excised with a 1 cm gross margin, and if necessary, a pedicled or free flap was used for closure of the defect. Selective (I–III/IV) neck dissection was performed for a cN0 neck and a comprehensive dissection (I–V) for cN+ disease. Adjuvant treatment was given for T3/4 disease, nodal metastases and/or adverse pathological features, as determined by a multidisciplinary team. We excluded patients with oropharyngeal cancer (who would be treated with an organ‐preserving strategy such as definitive radiotherapy), recurrent disease and metachronous cancers. An age cut‐off of 45 years was used to define young and old cohorts, as supported by existing literature. Patient demographics, clinicopathological features, adjuvant treatment and long‐term oncologic outcomes were collated from all centres.

Patients were restaged using the AJCC 8th edition (TNM) staging, with reporting at all centres performed by a dedicated head and neck pathologist. Histopathological reporting was based on strict adherence to internationally accepted protocols. Degree of differentiation was based on Broders' grading system while perineural invasion (PNI) and lymphovascular invasion (LVI) was reported when there was clear evidence of tumour infiltration involving at least one‐third of the nerve/vessel.^10^, ^11^ Histological margins were defined as clear (microscopically free margin >5 mm from tumour) or close/involved (when ≤5 mm from tumour).^10^ Institutional ethics committee approval was sought and written informed consent obtained before the study was commenced.

### 2.2 Loss of productivity economic analysis

A separate analysis exploring the economic cost of premature mortality among young patients with OSCC was performed in India and Singapore. This utilised national health, economic, labour and productivity data from the last 5 years (2015–2019) that was available.

To estimate the cost of premature cancer mortality in each country, the Human Capital Approach (HCA) was used. The HCA estimates the direct costs to society from a loss of productivity. It quantifies the loss of economic output from disease‐related morbidity and mortality when valued by market wage.^12^ Specifically, we estimated the Years of Potential Productive Life Lost (YPPLL), following the methodology described by O'Lorcain et al.^13^ YPPLL is the numerical difference between a country's official retirement age (62 for Singapore and 60 for India) and the age at which death occurred from OSCC. For example, when death from OSCC occurred at 40 years for a Singaporean, the YPPLL would be (62–40) 22 years of lost economic productivity. Younger patients who succumb to premature mortality have a higher YPPLL, and consequently, represent a greater economic loss to society. The number of deaths were compiled across 5‐year age brackets for both males and females in each country and multiplied by their YPPLL to give a cumulative YPPLL. Deaths in children (<15 years old), and adults beyond their country's retirement age were excluded, thereby capturing an economically active cohort.

Subsequently, we valued the cost of premature mortality by multiplying for each death, the YPPLL by age bracket, with gross wage. Estimates were adjusted for the age‐stratified unemployment rate, labour participation rate, wage growth rates and inflation (refer to Appendix S1). For example, in 2019, a 40‐year‐old Singaporean female would have an expected gross annual salary of US$ 40,261 (SG$ 54, 756), unemployment rate of 2.5% and a labour participation rate of 81.2%, within the 40 to 44‐year age bracket. Her total premature mortality cost from death would be US$ 1,084,079 ($40,260 × 22 years PPLL × 0.975 employment rate × 0.812 labour participation rate × 1.02^22^ wage growth). With this, we computed two outcome measures: the premature cost of mortality per death, and cost per YPPLL (US$), stratified by age group, gender and country.

In Singapore, wage growth was projected at 3% a year, with inflation at 1%.^14^ In India, annual wage growth was 9%, discounted by 5% for inflation.^15^ Costs were expressed in 2022 US Dollars (US$ 1: SG$ 1.37: 75 Indian Rupees). Absolute death rates stratified by 5‐year age brackets and gender for each country were obtained from the Global Burden of Disease Collaborative Network, Institute for Health Metrics and Evaluation (IHME).^16^ Labour, employment, median wage, inflation and wage growth rates in Singapore were provided by the Singapore Department of Statistics and derived from the Central Provident Fund.^14^ Similar data for India was obtained from the Reserve Bank of India, International Labour Organisation and World Bank Group Archives database.^15^, ^17^, ^18^ No ethics approval was required for this aspect of the study as the data are publicly available from online repositories.

### 2.3 Statistical analysis

All statistical analyses were performed using SPSS version 25 (Windows, IBM). Categorical variables were analysed with the Pearson *X* ^2^ or Fisher exact test, while continuous variables were analysed with a paired *t*‐test or Mann–Whitney *U* test. Survival estimates were calculated with the Kaplan–Meier method. Differences between survival curves were computed using the log‐rank test. Univariable analysis of factors predicting death and recurrence were analysed with logistic regression. All significant variables (with *p* < 0.05 on univariable analysis) were used to build a multivariable logistic regression model using backward selection. Hazard Ratios (HR) were generated for each stage‐specific survival curve, and 95% confidence intervals (95% CI) were reported alongside. Subgroup analysis was performed to study the effect of tobacco use on the survival of young patients with OSCC, using Cox regression. *p* value <0.05 was taken to be statistically significant. Complete case analysis was used for missing data.

## 3 RESULTS

### 3.1 Patient, disease and treatment characteristics

Two thousand one hundred and thirty‐eight patients with OSCC were analysed—478 belonged to a young cohort (<45 years old), while 1660 were older (≥45 years old). Median duration of follow‐up was 24 months (range 1–270). The median age of diagnosis of OSCC in the young cohort was 39 years (range 17–44) and 60 years (range 45–102) in the older group. Majority of patients were male (63.8%) and approximately 40% of each cohort used tobacco. Subsite involvement varied between groups, with a higher incidence of tongue cancer in the young versus old cohorts (62.1% vs. 51.5%), a similar distribution of buccal cancer (23.4% vs. 22%), fewer floor of mouth (6.9% vs. 10.7%) and alveolus/retromolar trigone cancers (5.4% vs. 12%). Staging between the two groups was comparable (*p* = 0.97). Of note, adverse histopathological features were more frequent in the young cohort—PNI 42.9% versus 35% (*p* = 0.002), LVI 22.4% versus 17.3% (*p* = 0.013), moderate/poor tumour differentiation 70.1% versus 65.2% and extranodal extension (ENE) 36% versus 24.5% (*p* < 0.001). Young patients received more aggressive adjuvant treatment—56.1% versus 49.5% received any form of treatment, and 25.9% versus 16.6% (*p* < 0.001) received concurrent chemoradiotherapy (CCRT) (Table 1).

**TABLE 1:** Clinicopathological factors for OSCC in young (<45 years) and old (≥45 years) patients.

Clinicopathological factor	Young (%) *n* = 478	Old (%) *n* = 1660	*p* Value
Gender			
Male	318 (66.5)	1047 (63.1)	0.17
Female	160 (33.5)	613 (36.9)	
Subsite			
Tongue	297 (62.1)	855 (51.5)	<0.001
Floor of mouth	33 (6.9)	177 (10.7)	
Buccal	112 (23.4)	366 (22.0)	
Alveolus/RMT	26 (5.4)	199 (12.0)	
Lip	3 (0.6)	28 (1.7)	
Hard palate	7 (1.5)	30 (1.8)	
Age			
Median, years (Range)	39 (17–44)	60 (45–102)	<0.001
Tobacco users			
Yes	185 (39.8)	640 (40.9)	0.65
Missing	13 (2.7)	97 (5.8)	
Histologic grade^a^			
WD	137 (29.8)	556 (34.7)	0.13
MD	276 (60.1)	885 (55.2)	
PD	46 (10.0)	161 (10.0)	
Mean tumour diameter, mm (SD)	29.6 (17.1)	28.6 (15.9)	0.04
Mean tumour thickness, mm (SD)	14.1 (10.8)	12.6 (10.2)	0.13
DOI^b^			
0 mm	1 (0.2)	6 (0.4)	0.27
1–5 mm	114 (24.2)	426 (27.0)	
6–10 mm	147 (31.1)	521 (33.0)	
>10 mm	210 (44.5)	625 (39.6)	
PNI^c^			
Yes	201 (42.9)	552 (35.0)	0.002
Missing	10 (2.1)	81 (4.9)	
LVI^d^			
Yes	104 (22.4)	274 (17.3)	0.013
Missing	14 (2.9)	79 (4.8)	
Bone invasion			
Yes	63 (13.4)	292 (17.8)	0.023
Neck dissection			
None	46 (10.9)	156 (10.7)	0.02
Selective	331 (78.3)	1065 (72.9)	
Comprehensive	46 (10.9)	239 (16.4)	
Extranodal extension			
Yes	143 (36.0)	329 (24.5)	<0.001
Margin involvement			
Clear >5 mm	383 (81.0)	1191 (73.5)	0.001
Near or involved <5 mm	90 (19.0)	429 (26.5)	
pT Stage			
T1	93 (19.5)	345 (20.8)	0.13
T2	137 (28.7)	488 (29.4)	
T3	115 (24.1)	319 (19.2)	
T4	133 (27.8)	508 (30.6)	
pN Stage			
N0	266 (55.6)	1018 (61.4)	0.006
N1	38 (7.9)	166 (10.0)	
N2	172 (36.0)	473 (28.5)	
N3	2 (0.4)	2 (0.1)	
AJCC 8 Stage			
Stage I	86 (18.0)	299 (18.0)	0.97
Stage II	97 (20.3)	329 (19.8)	
Stage III	73 (15.3)	269 (16.2)	
Stage IV	222 (46.4)	763 (46.0)	
Adjuvant treatment			
None	210 (43.9)	839 (50.5)	<0.001
RT	144 (30.1)	545 (32.8)	
ChemoRT	124 (25.9)	276 (16.6)	
Recurrence			
Yes	140 (29.3)	437 (26.3)	0.28
Locoregional recurrence			
Yes	53 (12.3)	187 (14.2)	0.34
Distant recurrence			
Yes	47 (10.9)	126 (9.5)	0.40

^a^ MD, moderately differentiated; PD, poorly differentiated; WD, well differentiated.

^b^ DOI, depth of invasion.

^c^ PNI, perineural invasion.

^d^ LVI, lymphovascular invasion.

### 3.2 Stage‐specific survival between young and old cohort

**FIGURE 1:**
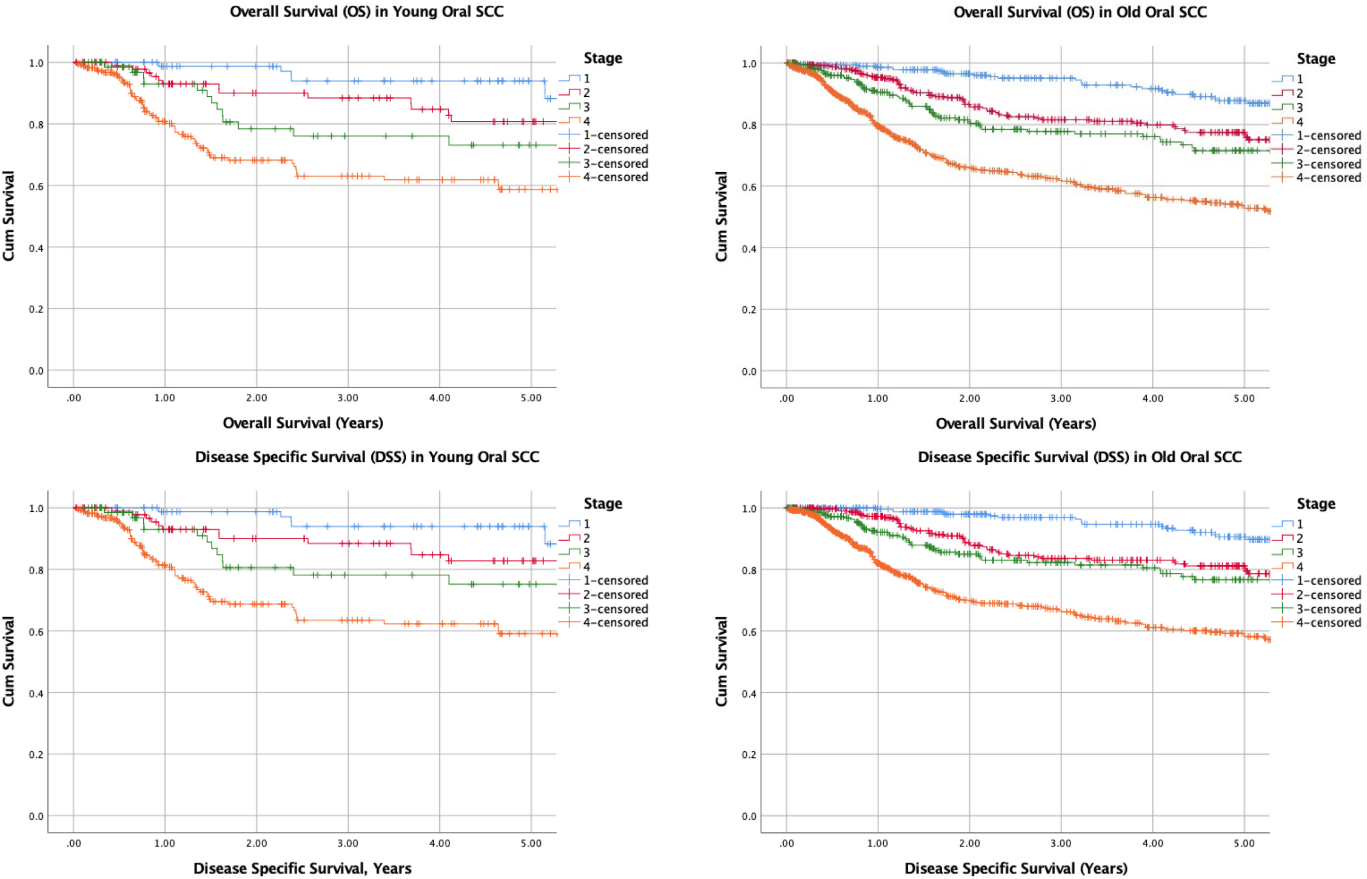
Five‐year overall survival (OS) and disease‐specific survival (DSS) for young versus old cohort.

Kaplan–Meier survival curves were plotted (Figure 1 and Figures S1–S3) to illustrate the TNM Stage‐specific 5‐year overall survival (OS), disease specific survival (DSS) and recurrence free survival (RFS) of each cohort. After considering a pathologically more aggressive disease in the young (from a higher incidence of adverse histological features) coupled with intensive adjuvant treatment, survival between both cohorts was similar. Five‐year OS were 93.9% versus 86.9% (*p* = 0.14) for Stage I, 80.7% versus 75% (*p* = 0.29) for Stage II, 73.1% versus 71.5% (*p* = 0.53) for Stage III and 58.7% versus 52.8% (*p* = 0.27) for Stage IV. Five‐year DSS were 88.2% versus 89.9% (*p* = 0.84) for Stage I, 82.7% versus 78.6% (*p* = 0.63) for Stage II, 75.1% versus 76.6% (*p* = 0.86) for Stage III and 59.1% versus 58.2% (*p* = 0.96) for Stage IV. Five‐year RFS was 90% versus 87.6% (*p* = 0.91) in Stage I, 73.8% versus 70.4% (*p* = 0.50) in Stage II, 64% versus 65.6% (*p* = 0.66) in Stage III and 46.4% versus 55.5% (*p* = 0.07) in Stage IV disease (Table 2).

**TABLE 2:** Five‐year overall survival (OS), disease‐specific survival (DSS) and recurrence‐free survival (RFS) in the young versus old cohort.

TNM Stage	Young	Old	*p* Value	Young
	5‐year OS (%)			
I	93.9	86.9	0.14	1.91 (0.80–4.53)
II	80.7	75.0	0.29	1.37 (0.77–2.46)
III	73.1	71.5	0.53	1.21 (0.67–2.22)
IV	58.7	52.8	0.27	1.17 (0.89–1.55)

TNM Stage	5‐year DSS (%)			
I	88.2	89.8	0.84	1.01 (0.44–2.73)
II	82.7	78.6	0.63	1.16 (0.63–2.16)
III	75.1	76.6	0.86	0.94 (0.50–1.79)
IV	59.1	58.2	0.96	0.99 (0.74–1.32)

TNM Stage	5‐year RFS (%)			
I	90.0	87.6	0.91	1.04 (0.54–1.98)
II	73.8	70.4	0.50	1.18 (0.73–1.90)
III	64.0	65.6	0.66	0.90 (0.55–1.47)
IV	46.4	55.5	0.07	0.80 (0.62–1.02)

### 3.3 Negative prognostic factors for recurrence and death in young patients

Logistic regression (LR) was used to investigate the factors driving recurrence and death amongst young patients with OSCC. On univariable LR, tobacco use, differentiation, PNI, LVI, bone invasion, depth of invasion (DOI), ENE, pT, pN and adjuvant treatment were negative prognostic factors for recurrence (*p* < 0.05). On multivariable L, tobacco use (*p* = 0.034), LVI (*p* = 0.02) and ENE (*p* = 0.03) were significant in predicting recurrence (Table 3).

**TABLE 3:** Logistic regression analysis for factors predictive of recurrence and death in the young cohort.

Variable	Univariate analysis		Multivariable analysis	
	Hazard ratio (95% CI)	*p* Value	Hazard ratio (95% CI)	*p* Value
Recurrence				
Gender	1.13 (0.92–1.38)	0.24	–	–
Tobacco use	1.22 (1.00–1.49)	0.048	1.66 (1.04–2.66)	0.034
Differentiation	1.48 (1.27–1.74)	< 0.001	1.46 (0.97–2.20)	0.71
PNI^a^	2.02 (1.66–2.47)	< 0.001	–	–
LVI^b^	2.84 (2.25–3.58)	< 0.001	1.87 (1.10–3.17)	0.02
Bone Invasion	1.30 (1.02–1.67)	0.038	–	–
DOI^c^	1.02 (1.01–1.03)	<0.001	1.41 (0.99–2.00)	0.057
ECS^d^	2.72 (2.17–3.41)	<0.001	1.77 (1.06–2.96)	0.03
Close/involved margins	1.19 (0.96–1.48)	0.12	–	–
pT	1.30 (1.19–1.42)	<0.001	–	–
pN	1.54 (1.39–1.71)	<0.001	–	–
Adjuvant RT	1.67 (1.34–2.09)	<0.001	–	–
Adjuvant CCRT	2.78 (2.16 0 3.57)	<0.001	–	–
Death				
Gender	1.01 (0.82–1.25)	0.91	–	–
Tobacco use	1.47 (1.20–1.81)	< 0.001	1.30 (1.04–1.63)	0.022
Differentiation	1.65 (1.40–1.95)	< 0.001	1.31 (1.08–1.59)	0.006
PNI	1.72 (1.39–2.12)	< 0.001	–	–
LVI	2.47 (1.94–3.13)	< 0.001	1.56 (1.18–2.06)	0.002
Bone invasion	1.59 (1.24–2.05)	< 0.001	–	–
DOI	1.03 (1.02–1.04)	< 0.001	–	–
Extranodal extension (ENE)	3.01 (2.37–3.83)	< 0.001	–	–
Close/involved margins	1.67 (1.34–2.09)	< 0.001	1.42 (1.10–1.83)	0.008
pT	1.36 (1.24–1.49)	< 0.001	1.13 (1.01–1.26)	0.038
pN	1.69 (1.52–1.88)	< 0.001	1.45 (1.26–1.65)	<0.001
Adjuvant RT	1.55 (1.12–1.95)	< 0.001	–	–
Adjuvant CCRT	1.87 (1.44–2.44)	< 0.001	–	–

^a^ PNI, perineural invasion.

^b^ LVI, lymphovascular invasion.

^c^ DOI, depth of invasion.

^d^ ECS, extra capsular spread.

Factors predictive of death on univariable LR include tobacco use, differentiation, PNI, LVI, bone invasion, DOI, ENE, close/involved margins, pT, pN and adjuvant treatment. On multivariable analysis, tobacco use (*p* = 0.022), differentiation (*p* = 0.006), LVI (*p* = 0.002), close/involved margins (*p* = 0.008), pT (*p* = 0.038) and pN (*p* < 0.001) predicted death.

### 3.4 Subgroup analysis

Of interest, tobacco use was found to be significant in predicting both death and recurrence on multivariable analysis. When stratified by tobacco use, young patients who were non‐smokers had a superior 5‐year OS and DSS as compared to smokers, particularly so for Stage II–IV disease. Five‐year OS (for non‐smokers vs. smokers) was 90.7% versus 96.7% for Stage I, 83.9% versus 73.4% for Stage II, 80.6% versus 60.2% for Stage III and 61.3% versus 58% for Stage IV (*p* < 0.001). For 5‐year DSS, survival rates were 90.7% versus 96.7% for Stage I, 87.1% versus 73.4% for Stage II, 84.6% versus 60.2% for Stage III and 61.3% versus 58.8% for Stage IV (*p* < 0.001) (Table 4).

**TABLE 4:** Five‐year overall survival (OS) and disease‐specific survival (DSS) in the young cohort stratified by smoking status.

OS	Non‐smoker	Smoker	*p* Value	HR (95% CI)
	5‐year OS (%)			
Stage I	90.7	96.7	< 0.001	1.64 (1.42–1.88)
Stage II	83.9	73.4	< 0.001	1.61 (1.37–1.88)
Stage III	80.6	60.2	< 0.001	1.74 (1.42–2.14)
Stage IV	61.3	58.0	< 0.001	1.86 (1.54–2.24)

DSS	5‐year DSS (%)			
Stage I	90.7	96.7	< 0.001	1.78 (1.52–2.06)
Stage II	87.1	73.4	< 0.001	1.72 (1.45–2.03)
Stage III	84.6	60.2	< 0.001	1.83 (1.47–2.29)
Stage IV	61.3	58.8	< 0.001	1.77 (1.46–2.14)

### 3.5 Cost of premature mortality and health economic analysis

**FIGURE 2:**
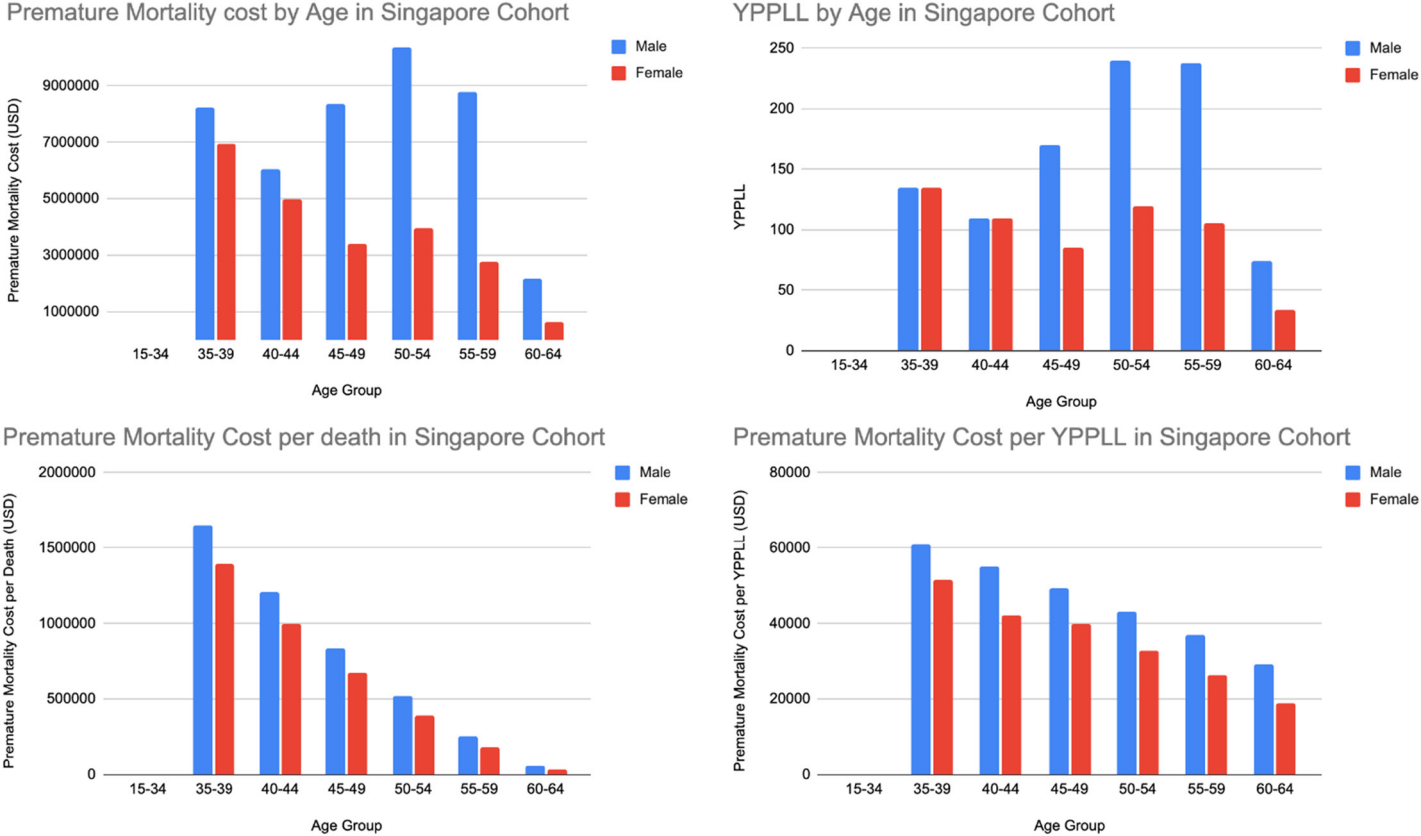
YPPLL and cost of premature mortality in Singapore.

**FIGURE 3:**
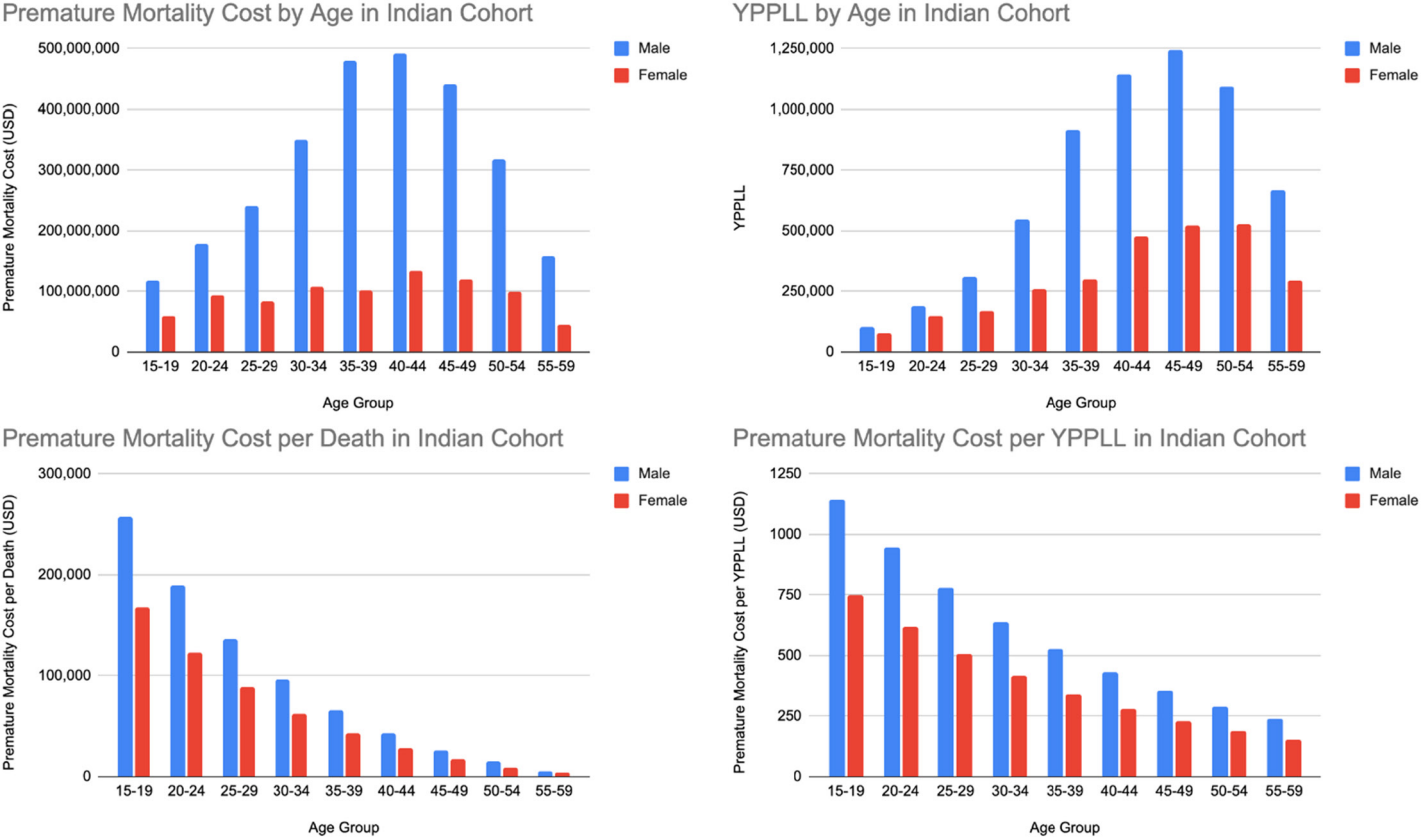
YPPLL and cost of premature mortality in India.

Figures 2 and 3 illustrate the number of deaths in 5‐year age brackets, their YPPLL and the cost of premature mortality for both genders in Singapore and India. Data were stratified by gender to reflect the differing workforce participation and unemployment rates. In Singapore, there were a total of 111 male and 57 female deaths among the economically active population between 2015 and 2019. For males, this represented 967 YPPLL (years) lost to society and is valued at US$ 43,984,628 in economic loss. For females, 589 YPPLL (years) were lost, amounting to US$ 22,651,932. In total, the potential economic loss to Singapore from early mortality from OSCC was US$ 66,636,560. The average premature mortality cost per death was US$ 396,258, and US$ 45,486 per YPPLL lost for males, while that for females was US$ 397,402 per death, and US$ 38,458 per YPPLL (refer to Tables S1 and S2).

In India, there were a total of 90,705 male and 40,273 female deaths between 2015 and 2019. For males, 6,211,550 YPPLL (years) were lost, resulting in a US$ 2,779,311,848 economic loss from premature death. For females, 40,273 YPPLL (years) were lost, amounting to US$ 847,283,149. The total economic loss from premature death was US$ 3,626,594,997. The average premature mortality cost per death was US$ 30,641 and US$ 595 per YPPLL for males, while that for females was US$ 21,038 per death, and US$ 305 per YPPLL.

## 4 DISCUSSION

Tobacco use and alcohol consumption have been well established in the carcinogenesis of elderly‐onset OSCC. This typically requires decades of chronic exposure for the accumulation of sufficient genetic mutations and ‘field cancerisation’ of the oral cavity mucosa.^19^, ^20^ Young‐onset OSCC, however, lacks the time for this multistep process of carcinogenesis, suggesting other elements are at play. With the growing incidence of OSCC in the young and its devastating socio‐economic consequences, there has been keen interest in comparing outcomes with the traditionally older cohort by postulating differences in genetic mutations, oral microbiome, immune microenvironment and the cellular processing of carcinogens. Uncovering these differences may influence prognosis, treatment response and even escalation of therapy.^5^, ^6^, ^7^, ^8^

Our study had a two‐pronged focus: a clinical analysis of oncologic outcomes between young versus old OSCC, and its wider application to society in terms of economic loss (or cost). When stratified by age, we found interesting differences between both cohorts. Adverse histopathological features, in particular, PNI (42.9% vs. 35%, *p* = 0.002), LVI (22.4% vs. 17.3%, *p* = 0.013) and ENE (36% vs. 24.5%, *p* < 0.001) were more prevalent in the young, asserting that young‐onset OSCC is a pathologically more aggressive disease. This is supported by our logistic regression model—where LVI and ENE were independent negative prognostic factors for recurrence, while LVI and poor tumour differentiation were predictive of poorer survival. Although the impact of these adverse features on survival is controversial in the literature, much of this stems from heterogeneity in the definition of PNI/LVI between centres and their indications for adjuvant radiotherapy.^21^ Our group harmonised the definitions of PNI/LVI amongst pathologists across all institutions, and previously concluded that the cumulation in number of these adverse features did contribute to a stepwise deterioration in survival.^22^ Younger patients, with their resilient functional reserves and fewer medical comorbidities, are also better able to tolerate and receive intensive adjuvant therapy. Of the young cohort, 25.9% received CCRT, while only 16.6% in the old cohort did so (*p* < 0.001). We postulate that the increased ability to deliver intensive adjuvant treatment in younger patients had a role in mitigating the effects of adverse pathological features. Elderly patients also are more likely to have other medical comorbidities, which may limit their ability to complete intensive adjuvant treatment, and in turn, adversely affect survival. These are plausible explanations for why 5‐year OS, DSS and RFS were similar between both cohorts. Within the literature, there is a divide on these outcomes.^23^, ^24^, ^25^, ^26^, ^27^, ^28^ A meta‐analysis by Panda et al evaluating 4981 patients across 25 studies concurred that young‐onset disease was pathologically more aggressive. Higher recurrence rates, distal metastases and a poorer DFS were seen in the young. Interestingly, OS was found to be superior in the younger cohort. However, this study used 40 years as an age cut‐off between young and old onset disease, included oropharyngeal cancers and had at least a moderate level of clinical and statistical heterogeneity between studies.^23^ The meta‐analysis by Lee et al, comprising 23,382 patients across nine studies, found similar OS and DFS trends. Although they showed similar results with a cut‐off at 40 years and after a supplemental analysis at 45 years, 80% of patients had early T1‐2 OSCC, likely accounting for the similarities in long‐term oncologic outcomes from early‐stage disease.^24^ Most studies agree that OS is similar between groups, but recurrence rates tend to be higher in the young.^25^, ^26^, ^27^, ^29^

We found that tobacco use was predictive of poorer survival and higher recurrence on multivariable analysis. Young non‐smokers formed a distinct epidemiological subset, with significantly improved OS, DSS, and had a favourable prognosis as compared to smokers. Yang et al evaluated a Chinese cohort of young OSCC, concluding that non‐smokers had superior locoregional control and improved DSS. Multiple theories have been proposed to explain this, such as a lower Ki‐67 proliferation index, p53 and p63 expression in non‐smokers.^5^, ^30^ Of interest, our cohort had a high proportion of non‐smokers, comprising almost 60% of patients, mostly from Kerala and Singapore. This is unlike what we see in most studies evaluating OSCC and brings a new dimension to the available literature.

The OSCC ‘belt’ stretches across continental Asia and Southeast Asia. India accounts for a third of the global OSCC burden, with 77,000 new cases and 52,000 deaths annually.^31^ In Singapore, OSCC accounts for approximately 4% of all cancers, with 232 cases diagnosed annually.^32^ While the absolute numbers are vastly different between the two countries, these results should be interpreted taking into consideration each country's disease prevalence and population size, which was 5,600,000 (2,600,000 economically active workforce) in Singapore, and 1,417,173,000 for India in 2022. The prevalence of OSCC is higher in India, affecting 21.7 per 100,000 persons, while in Singapore it is 9.8 per 100,000.^14^, ^16^, ^18^ From our health economic model focusing on the active workforce, 1556 YPPLL (years) were lost to society, valued at US$ 66.6 Million over 5 years (2015–2019). Each premature death cost the country almost US$ 400,000. In India, 8,990,200 YPPLL were lost, totalling US$ 3.6 billion, in keeping with her much larger population and prevalence of OSCC. We reported country specific figures for males and females separately, further stratifying them by 5‐year age brackets. This was due to the varying unemployment, labour participation and gross wage rates, which vary with age, gender and country. While gross incomes and labour participation rates were much lower in India as compared to Singapore, the sheer volume of OSCC cases was responsible for the differences in total economic loss. The outcome measures of premature mortality cost per YPPLL and cost per death add further granularity to the comparison between countries. As cancer research evolves from reporting the traditional oncologic outcomes, we have seen a rise in studies analysing Quality of Life and other nontangible measures. The loss of productive lifespan is one such measure that has rarely been studied or reported and is becoming an important theme in cancer research.^33^ To our knowledge, this is the first paper enumerating the cost of premature mortality per death and per YPPLL in the literature for OSCC. While OS may be similar between the young and old cohort, the early loss of life and economic cost at 40 years old is invariably much higher than for a 70‐year‐old patient. Hence, this represents an important socio‐economic outcome measure, which is country and context specific, and is growing in relevance.

Strength wise, this was an international collaborative effort across multiple tertiary referral centres specialising in the management of head and neck cancer. It comprised of a large cohort (2138 patients), with standardised definitions in pathological reporting across centres, thus enhancing the internal validity of the presented data. While many studies in the literature are limited to specific subsites such as tongue or buccal mucosa only, we presented results across all subsites, improving our results' generalisability to the wider population. Our second focus on the devasting health economic consequences of premature mortality from OSCC is a novel analysis which to our knowledge, has never been modelled in these countries before. The differences in incidence of OSCC, labour productivity rates, wage, inflation and retirement ages between countries suggest that there is significant heterogeneity in economic analyses between countries. To paint an accurate picture, such actuarial analyses have to be context and country specific, to show the gravity of premature mortality and its cost to society. As such, we modelled Singapore and India separately, using robust population‐level data, a validated methodology to value lost productivity, and captured the economic cost of premature mortality by focusing only on the active workforce, rather than the entire population. The adopted model is a novel idea in the scene of OSCC and can be easily reproduced for any country and malignancy. This powerful yet simple tool can elucidate the socio‐economic and productivity losses for any cancer. We believe this has widespread implications in the public health scene. Premature mortality and loss of productivity analysis facilitates tailoring of early cancer screening and educational outreach campaigns. At the healthcare systems and policymaker level, these findings should be leveraged to divert funding towards cancers with a high socio‐economic disease burden. For example, educational campaigns to discourage tobacco and betel nut use from a young age should be expanded in India. With the growing trend in early‐onset oral cancer, policy changes should be implemented for earlier screening. From the translational point of view, more funding is needed for cancer genetic research, developing novel biomarkers and improving the efficacy of adjuvant treatment.

Our retrospective and economic analysis also has limitations. The cut‐off age of 45 years defining young and old cohorts is an arbitrary one. Previous studies included cut‐offs ranging from 25 to 45 years, and this nonuniformity between studies is an important contributor to heterogeneity in outcomes. Forty‐five years was the most consistent age adopted in the literature.^34^, ^35^ This is further supported by a genomic analysis by Marchiano et al, identifying crucial shifts in risk factor exposure, phenotypic and genotypic inflexion points at this age.^36^ A possible explanation for similarity in OS patterns between the young and old is that the latter could have a higher disease burden from comorbidities, which may discourage the use of aggressive adjuvant treatment. This may negatively affect the older cohort's OS and mask differences with the younger onset group. Our study, like others in the literature, was unable to account for the effect of comorbidities and was prone to missing data. The cohort studied comprised Singaporean and Indian patients treated in tertiary‐level Head and Neck Centres in major cities, which may not be generalisable to the wider population. With regards to the health economic analysis, only direct costs to society were measured. Nonmarket activities were excluded from our model. Household activities, such as time spent caregiving for the young and elderly, do have an indirect societal cost, but are difficult to quantify objectively. The assumptions in our model were based on labour and productivity data from 2015 to 2019, which may change with time. This includes changes in labour and market conditions, such as the Consumer Price Index, inflation, wage growth rates and an ever‐increasing retirement age. We also did not estimate healthcare expenses and productivity losses from morbidity of disease, due to a lack of reliable data. The true cost of economic productivity is thus underestimated.

## 5 CONCLUSION

Young OSCC is a pathologically more aggressive disease, though its adverse effects on survival are mitigated by treatment intensification. While survival outcomes are not worse, the loss of life from early‐onset disease has a greater socio‐economic impact than in the elderly, from a major loss in economic productivity and societal involvement. These are major concerns especially in developing nations. More needs to be done to elucidate the genetic basis of oncogenesis, develop novel therapeutic agents, improve screening and the efficacy of intensive adjuvant treatment for this cohort of patients.

## AUTHOR CONTRIBUTIONS

**Manraj Singh:** Conceptualization (lead); data curation (lead); formal analysis (lead); methodology (equal); project administration (equal); validation (equal); writing – original draft (lead); writing – review and editing (lead). **Krishnakumar Thankappan:** Conceptualization (equal); data curation (equal); project administration (equal); resources (equal); supervision (equal); validation (equal); writing – review and editing (equal). **Deepak Balasubramanian:** Conceptualization (equal); data curation (equal); investigation (equal); methodology (equal); project administration (equal); resources (equal); supervision (equal); validation (equal); writing – review and editing (equal). **Vijay Pillai:** Conceptualization (equal); data curation (equal); investigation (equal); methodology (equal); project administration (equal); resources (equal); supervision (equal); validation (equal); writing – review and editing (equal). **Vivek Shetty:** Conceptualization (equal); data curation (equal); investigation (equal); methodology (equal); project administration (equal); resources (equal); supervision (equal); validation (equal); writing – review and editing (equal). **Vidyabhushan Rangappa:** Conceptualization (equal); data curation (equal); investigation (equal); methodology (equal); project administration (equal); resources (equal); supervision (equal); validation (equal); writing – review and editing (equal). **Naveen Hedne Chandrasekhar:** Conceptualization (equal); data curation (equal); investigation (equal); methodology (equal); project administration (equal); resources (equal); supervision (equal); validation (equal); writing – review and editing (equal). **Vikram Kekatpure:** Conceptualization (equal); data curation (equal); investigation (equal); methodology (equal); project administration (equal); resources (equal); supervision (equal); validation (equal); writing – review and editing (equal). **Moni Abraham Kuriakose:** Conceptualization (equal); data curation (equal); investigation (equal); methodology (equal); project administration (equal); resources (equal); supervision (equal); validation (equal); writing – review and editing (equal). **Arvind Krishnamurthy:** Conceptualization (equal); data curation (equal); investigation (equal); methodology (equal); project administration (equal); resources (equal); supervision (equal); validation (equal); writing – review and editing (equal). **Arun Mitra:** Conceptualization (equal); data curation (equal); investigation (equal); methodology (equal); project administration (equal); resources (equal); supervision (equal); validation (equal); writing – review and editing (equal). **Arun Pattatheyil:** Conceptualization (equal); data curation (equal); investigation (equal); methodology (equal); project administration (equal); resources (equal); supervision (equal); validation (equal); writing – review and editing (equal). **Prateek Jain:** Conceptualization (equal); data curation (equal); investigation (equal); methodology (equal); project administration (equal); resources (equal); supervision (equal); validation (equal); writing – review and editing (equal). **Subramania Iyer:** Conceptualization (equal); data curation (equal); investigation (equal); methodology (equal); project administration (equal); resources (equal); supervision (equal); validation (equal); writing – review and editing (equal). **N. Gopalakrishna Iyer:** Conceptualization (equal); data curation (equal); formal analysis (lead); investigation (lead); methodology (equal); project administration (equal); resources (equal); supervision (equal); validation (equal); writing – original draft (lead); writing – review and editing (lead). **Narayana Subramaniam:** Conceptualization (lead); data curation (lead); formal analysis (lead); investigation (lead); methodology (lead); project administration (equal); resources (equal); supervision (equal); validation (equal); writing – original draft (lead); writing – review and editing (lead).

## FUNDING INFORMATION

No financial support was taken for this article.

## CONFLICT OF INTEREST STATEMENT

There are no financial disclosures or conflicts of interest for any of the authors.

## Supporting information


Appendix S1

## Data Availability

The datasets during and/or analysed during the current study are available from the corresponding author on reasonable request.
