# Supplementary material for: Contrasting clinical outcomes and socio‐economic impact of young versus elderly‐onset oral squamous cell carcinoma, a novel health economic analysis
Source: Cancer Med. 2024 Jan 15;13(3):e6747. doi: 10.1002/cam4.6747 (PMC10905235; doi:10.1002/cam4.6747)
Supplement: Supplementary file 1 — Appendix S1 [file CAM4-13-e6747-s001.docx]

Supplementary Table 1. Number of deaths, YPPLL and premature cost of mortality in Singapore and India stratified by gender and age

| Age Group | Deaths | YPPLL | Premature Mortality Cost | Deaths | YPPLL | Premature Mortality Cost |
| --- | --- | --- | --- | --- | --- | --- |
| Singapore | **Male** | | | **Female** |  |  |
| 15-34  35-39  40-44  45-49  50-54  55-59  60-64  Total | 0  5  5  10  20  34  37  111 | 0  135  110  170  240  238  74  967 | 0  8,241,090  6,042,569  8,375,970  10,387,646  8,772,333  2,165,020  43,984,628 | 0  5  5  5  10  15  17  57 | 0  135  110  85  120  105  34  589 | 0  6,949,761  4,980,261  3,387,918  3,938,326  2,752,763  642,903  22,651,932 |
| India  15-19  20-24  25-29  30-34  35-39  40-44  45-49  50-54  55-59  Total | **Male**  459  943  1769  3657  7312  11,414  16,613  21,869  26,669  90,705 | 103,275  188,600  309,575  548,550  914,000  1,141,400  1,245,975  1,093,450  666,725  6,211,550 | 118,327,086  178,634,989  241,040,644  350,752,684  480,208,200  492,267,617  440,746,587  317,935,789  159,398,252  2,779,311,848 | **Female**  352  754  952  1,734  2,399  4,772  6,944  10,597  11,769  40,273 | 79,200  150,800  166,600  260,100  299,875  477,200  520,800  529,850  294,225  2,778,650 | 59,218,948  93,036,224  84,379,379  108,180,769  102,528,987  133,762,789  120,054,754  100,259,895  45,861,403  847,283,149 |

Supplementary Table 2. Premature mortality cost per death & per YPPLL in Singapore and India, stratified by gender and age

| Age Group | Premature Mortality Cost per death (USD) | Premature Mortality Cost per YPPLL (USD) | Premature Mortality Cost per death (USD) | Premature Mortality Cost per YPPLL (USD) |
| --- | --- | --- | --- | --- |
| Singapore | **Male** |  | **Female** |  |
| 15-34  35-39  40-44  45-49  50-54  55-59  60-64  Average | 0  1,648,218  1,208,514  837,597  519,382  258,010  58,514  396,258 | 0  61,045  54,932  49,270  43,282  36,859  29,257  45,486 | 0  1,389,952  996,052  677,584  393,833  183,518  37,818  397,402 | 0  51,480  42,275  39,858  32,819  26,217  18,909  38,458 |
| India  15-19  20-24  25-29  30-34  35-39  40-44  45-49  50-54  55-59  Average | **Male**  257,793  189,433  136,258  95,913  65,674  43,128  26,530  14,538  5,977  30,641 | 1,146  947  779  639  525  432  354  291  239  595 | **Female**  168,236  123,390  88,634  62,388  42,738  28,031  17,289  9,461  3,897  21,038 | 748  617  506  416  342  280  231  189  156  305 |

Supplementary Figure 1. 5-Year Overall Survival (OS) for young vs. old cohort

Supplementary Figure 2. 5-Year Disease Specific Survival (DSS) for young vs. old cohort

Supplementary Figure 3. 5-Year Recurrence-Free Survival (RFS) for young vs. old cohort

Supplementary Formula 1. The Human Capital Approach formula for the cost of premature mortality

YPPLL (years) = Country’s retirement age – age of death

Premature Mortality Cost = YPPLL x Annual wage (US$) x Unemployment rate (%) x Labour participation rate (%) x Wage growth rate^YPPLL^ (%)
